# Sexual Function in Women With Polycystic Ovary Syndrome: Design of an Observational Prospective Multicenter Case Control Study

**DOI:** 10.1016/j.esxm.2020.07.002

**Published:** 2020-08-12

**Authors:** Hester Pastoor, Stephanie Both, Reinier Timman, Ellen T.M. Laan, Joop S.E. Laven

**Affiliations:** 1 Division of Reproductive Endocrinology and Infertility, Department of Obstetrics and Gynecology, Erasmus University Medical Center, Rotterdam, the Netherlands; 2 Department of Psychosomatic Gynecology and Sexology, Leiden University Medical Center, Leiden, the Netherlands; 3 Department of Psychiatry, Section of Medical Psychology and Psychotherapy, Erasmus University Medical Centre, Rotterdam, the Netherlands; 4 Department of Sexology and Psychosomatic OBGYN, Amsterdam University Medical Centers, University of Amsterdam, Amsterdam, the Netherlands

**Keywords:** PCOS, Polycystic Ovary Syndrome, Female Sexual Function, Female Sexual Dysfunction, Photoplethysmography, Androgens

## Abstract

**Introduction:**

The prevalence of polycystic ovary syndrome (PCOS) is 10–15% in women of reproductive age. Its characteristics are (i) clinical or biochemical hyperandrogenism, (ii) oligomenorrhea or amenorrhea, and (iii) polycystic ovaries on ultrasound. PCOS is associated with lower quality of life, depression, anxiety, diabetes, and cardiovascular disease. Treatment commonly entails oral contraceptive use to lower endogenous androgen levels. Androgen levels and comorbidities may affect sexual function. Previous studies have addressed a limited range of possible contributing factors. We will assess sexual function as well as genital and self-reported sexual arousal in a laboratory setting in women with PCOS compared to an age-matched healthy control group. Modulation by biopsychosocial factors mentioned will be studied.

**Methods:**

This is a multicenter prospective case control study. The study population includes healthy women with and without PCOS, aged 18–40 years, in a stable heterosexual relationship for at least 6 months. Power is calculated at 67 participants in each group. Anticipating a drop out of 10%, 150 participants will be recruited.

**Main outcome measures:**

The main outcomes measured are sexual function using the Female Sexual Function Index, Sexual Desire Inventory, and Female Sexual Distress Scale-Revised; genital sexual arousal measured as vaginal pulse amplitude; and self-reported sexual arousal in response to erotic stimuli in a laboratory setting. The mediators that will be investigated include testosterone, free androgen levels, oral contraceptive use, sensitivity to androgens (using CAG repeat length), body mass index, body image, mental health, and self-esteem.

**Conclusion:**

Strengths of this study are the inclusion of a broad range of biopsychosocial outcome measures including DNA analysis, a healthy control group, and standardized assessment of genital and self-reported sexual arousal in a laboratory setting. With the design of this study we aim to provide an insight into which biopsychosocial factors associated with PCOS are related to sexual function, and how sexual function may be affected by treatment. These new insights may help to improve clinical management of PCOS while improving the quality of life.

**Pastoor H, Both S, Timman R, et al. Sexual Function in Women With Polycystic Ovary Syndrome: Design of an Observational Prospective Multicenter Case Control Study. Sex Med 2020;8:718–729.**

## Background

Polycystic ovary syndrome (PCOS) is the most common endocrine disease in women. Its prevalence is estimated to be between 5% and 15%^1^ in women of reproductive age. Its characteristics are (i) either clinical (hirsutism) or biochemical hyperandrogenism (elevated androgen serum levels), (ii) oligomenorrhea or amenorrhea, and (iii) polycystic ovaries on ultrasound.^2^ Following the Rotterdam criteria, PCOS is diagnosed when 2 out of 3 characteristics are present.^2^ Treatment of PCOS is complex and varies depending on symptoms and whether there is a desire to have children. In the latter group, the first-line treatment in women who are overweight or obese is lifestyle modification followed by ovulation induction. Lifestyle changes contribute to optimizing success rates in establishing a pregnancy and reducing complication rates by normalizing insulin resistance and consequently androgen levels.^3^, ^4^, ^5^, ^6^ In women who do not want to become pregnant, treatment usually consists of oral contraceptive pill (OCP) use and lifestyle changes if indicated.^7^ Both interventions aim at improving endocrine disturbances by normalizing insulin resistance and androgen metabolism. Similarly, OCPs increase SHBG levels and hence reduce free androgen levels.

PCOS is a distressing disease with symptoms such as subfertility, hirsutism, and acne.^7^, ^8^, ^9^, ^10^ It is associated with obesity, insulin resistance, and unfavorable lipid profiles.^7^^,^^9^ Also, PCOS is associated with lower quality of life, depression, and anxiety.^7^^,^^11^, ^12^, ^13^, ^14^, ^15^, ^16^ The most recently published evidence-based guideline on PCOS mentions reduced health-related quality of life scores for women with PCOS compared to a control population.^17^ Health-related quality of life is influenced by the clinical features of PCOS (mainly hirsutism, menstruation, and fertility) and affected by anxiety, poor body image, low self-esteem, depressive symptoms, delayed diagnosis, and inadequate education about PCOS. The guideline also reports a 3 times higher prevalence of depression^15^^,^^18^, ^19^, ^20^, ^21^, ^22^ and a 5-fold increase in the incidence of anxiety disorders^15^^,^^18^, ^19^, ^20^, ^21^, ^22^, ^23^, ^24^ in women with PCOS. Also, body image seems to be impaired in women with PCOS compared to a control population.^25^ Although there is conflicting evidence, hirsutism,^26^, ^27^, ^28^ increased weight,^27^^,^^28^ and infertility^26^ seem to affect body image negatively. A negative body image is strongly associated with depression.^27^^,^^29^^,^^30^ Finally, disordered eating, eating disorders (mainly bulimia nervosa), and risk factors for eating disorders seem to be more prevalent in women with PCOS.^19^^,^^31^, ^32^, ^33^ Psychosocial factors such as anxiety, depression,^34^^,^^35^ poor body image,^36^ and eating disorders^37^ are recognized as potential risk factors for sexual dysfunction and impaired sexual satisfaction.

Data on sexual function in women with PCOS are limited. A systematic review on PCOS and sexual function by our research group compared 18 studies in a meta-analysis.^38^ Sexual dysfunction appeared to be more prevalent in women with PCOS than in the control group. Particularly, sexual arousal, lubrication, orgasm, and satisfaction were compromised. Effect sizes were small for the first 3 aspects of sexual function; for satisfaction the effect size was large. In addition, women with PCOS have fewer sexual thoughts, report self-perceived impaired attractiveness, and report more problems participating in social meetings due to bodily appearance than women without PCOS. Also, women with PCOS report a negative impact of excessive body hair on sexual function. For these variables effect sizes were large, with the exception of number of sexual thoughts which showed a medium effect size. Although women with PCOS were less satisfied with their sex life, having a satisfying sex life was as important to them as it was for control women. In the studies assessed in this meta-analysis, sexual function was mainly studied using questionnaires. Sample sizes were too small to make subgroup comparisons to determine associations between sexual function and weight, hirsutism, mental health, body image, menstrual cycle, endocrine features, or use of OCPs.

It is known that sexual function is influenced by biological,^39^, ^40^, ^41^, ^42^, ^43^, ^44^, ^45^, ^46^ psychological,^34^, ^35^, ^36^^,^^47^, ^48^, ^49^ and social factors.^50^^,^^51^ As mentioned, in women with PCOS all these factors might be compromised,^17^ potentially leading to compromised sexual function.

Sex steroid hormones, in particular estrogens and androgens, play an important role in sexual function,^52^, ^53^, ^54^, ^55^, ^56^ although the exact mechanisms by which these steroids exert their influence are still unclear.^52^^,^^55^, ^56^, ^57^, ^58^, ^59^ Androgens are believed to sensitize the brain and genitals to sexual stimuli.^53^^,^^55^^,^^60^ Estrogens seem to improve sexual function indirectly by improving vaginal health and mood.^55^^,^^57^ Reduced levels of estrogens and androgens are associated with alterations in genital tissue structure and innervation, as well as with response to physiological modulators like neuropeptides and neurotransmitters.^61^ Furthermore, estrogen and androgen deficiencies are associated with reduced expression of sex steroid receptors and most importantly with attenuated genital blood flow and lubrication in response to pelvic nerve stimulation.^61^

An important feature of PCOS is the alteration in sex steroid hormones. In PCOS androgen levels are often elevated, leading to biochemical and/or clinical hyperandrogenism. However, women with PCOS do not report a higher level of sexual desire compared to women without PCOS.^38^ In general, the relationship between androgen levels and sexual desire in women is inconclusive.^57^ Partly, this may be due to the fact that many studies have assumed that androgens and sexual outcomes are linearly and cross-sectionally related across the total serum testosterone or free testosterone range. It is possible though that, as in men,^62^^,^^63^ androgen-related sexual problems in women should only be expected when androgen levels are below a certain hypophysiological threshold. Even less is known about sexual function in women who have androgen levels at the high end of the normal range or who have hyperphysiological androgen levels. In women, findings of a number of studies suggest a threshold approach might be more suitable.^64^, ^65^, ^66^, ^67^, ^68^, ^69^, ^70^, ^71^ This research project aims to contribute to this discussion.

Differences in androgen receptor (AR) activity and hence sensitivity to androgens might complicate the relationship between androgens and sexual function. Functional studies have demonstrated that an inverse relationship exists between the CAG repeat length and receptor expression impacting on the strength of the androgen action and sensitivity to sex hormones.^72^ Several authors have studied the potential association between shorter CAG repeats and a high testosterone level in PCOS as well as the general population, with conflicting results.^73^, ^74^, ^75^, ^76^, ^77^, ^78^, ^79^ Ethnicity, small sample sizes, and different definitions of PCOS and hyperandrogenism may underlie these inconsistent results. Moreover, a non-linear relationship between CAG length and AR activity has been suggested.^80^ Therefore, it is plausible that CAG repeat length variants are involved in the pathogenesis of women with hyperandrogenism and influence sexual function. For instance, a recent Danish population study showed that lower numbers of CAG repeats were associated with problems in reaching orgasm.^81^

The impact of OCP use on endocrine and sexual function has been studied extensively in healthy women.^82^, ^83^, ^84^, ^85^, ^86^, ^87^, ^88^, ^89^, ^90^, ^91^, ^92^ OCPs are known to decrease androgen and free testosterone levels.^56^^,^^57^^,^^90^^,^^92^ Hypothetically, sexual function can be influenced by this mechanism. Repeatedly, studies have shown that some women report an OCP-related improvement in sexual function, some a deterioration, whereas many report no change in sexual function.^82^, ^83^, ^84^, ^85^, ^86^^,^^88^^,^^89^^,^^93^ In women with PCOS, OCPs can restore androgen levels to normal, improving clinical symptoms like acne and to a lesser extent hirsutism.^7^ Few studies have assessed its influence on sexual function. Results varied from improvement of sexual function, while normalizing androgen levels,^94^ to deterioration of sexual desire.^95^

In addition to differences in androgen sensitivity, in women with PCOS neuronal circuits in the brain might be impaired due to hyperandrogenism, resulting in a disruption of sex steroid feedback mechanisms.^96^^,^^97^ Following this line of reasoning, we will assess androgen levels, CAG repeat length, and actual genital response to sexual stimuli (vibrotactile, film fantasy) in a psychophysiological laboratory setting while taking into account the other factors presented here. As a result, we hope to be able to shed more light on the role of androgen levels in sexual responsiveness of women with PCOS.

Women with PCOS report significantly less sexual arousal and lubrication compared to normal controls,^38^ but it is unknown whether women with PCOS actually show abnormal physiological sexual responses. A pilot study concerning the effect of circulating androgen levels found no significant differences in clitoral vascularization in non-aroused women with PCOS in comparison with control women,^98^ but nothing is known about the genital response to sexual stimulation. There are indications that androgens influence sexual response to sexual fantasies and genital tactile stimulation, whereas the effect of visual stimuli is less influenced by androgen levels.^99^^,^^100^ To objectively assess genital response to sexual stimuli and its contribution to sexual function, psychophysiological measurements with vaginal pulse amplitude (VPA) are required.

The primary aim of this study is to assess differences between women with PCOS and control women in the following sexual outcome measures: Female Sexual Function Index (FSFI), Female Sexual Distress Scale-Revised (FSDS-R), Sexual Desire Inventory (SDI), and genital and subjective sexual responsiveness to sexual stimuli measured with VPA. Secondary aims are to investigate differences in these outcome measures between OCP users and non-users in interaction with PCOS status in an exploratory way, and to assess factors that may mediate the relationship between PCOS status and sexual outcome measures.

We will assess the following mediators: testosterone, free androgen levels, OCP use, sensitivity to androgens (using CAG repeat length), body mass index (BMI), body image, mental health, and self-esteem.

We expect to find lower sexual function and higher sexual distress in the PCOS group compared with the control group.^38^ Further, we expect to find stronger genital and self-reported sexual responsiveness in women with higher androgen levels in both the fantasy and vibrotactile stimulation conditions but not with visual sexual stimuli.^99^^,^^100^

Following the threshold model,^64^, ^65^, ^66^, ^67^, ^68^, ^69^, ^70^, ^71^ we do not predict a linear relationship between VPA response and androgen levels (free testosterone and total testosterone). We do expect women with the lowest bioavailable androgen levels (control women using OCPs) to show lower levels of VPA response than women in the upper 3 quartiles of bioavailable androgen levels (women with PCOS not using OCPs).

We hypothesize that women with PCOS will score lower on various psychosocial and mental health measures compared with control women.^17^ We expect these scores to be associated with impaired scores on sexual function questionnaires.^34^, ^35^, ^36^, ^37^

We have no specific expectation for the relationship between sexual outcomes and CAG repeat length in both women with and without PCOS. CAG repeat length will be measured to exploratively assess individual differences in sensitivity to androgens and the relationship with PCOS status and other variables.

## Methods

### Design

This study is a non-randomized observational prospective case control study in a multicenter setting assessing the difference in sexual function between women with and without PCOS. Additionally, we examine the effect of OCP use resulting in a 2×2 design. The duration of the study will be 4 years. 3 Dutch academic medical centers will participate in this study: Erasmus Medical Center Rotterdam, Leiden University Medical Center (LUMC), and Amsterdam University Medical Center (AMC). Independent variables are PCOS status and OCP use. The dependent variables are sexual function as assessed with questionnaires and genital and self-reported sexual arousal in a laboratory setting.

Ethical approval was awarded by the Ethics Committee of Erasmus Medical Center, Rotterdam, The Netherlands (MEC 2016-216, NL55484.078.16). Local ethical approval was awarded by the Ethics Committees of LUMC (P16.299) and AMC (2016_182/NL55484.078.16).

### Participant Recruitment and Selection

Women who have been diagnosed with PCOS at the outpatient clinic of the Erasmus University Medical Center will be asked to participate in this study. Also, Dutch patient societies will be requested to advertise for our study. PCOS will be diagnosed according to the Rotterdam criteria.

An age-matched control group of healthy women will be recruited through advertisements in local newspapers, social media, or flyers at the outpatient gynecological clinics of Erasmus University Medical Center, LUMC, and AMC. Control women will also be recruited through the pool of volunteers available at LUMC. In the control group, women with PCOS will be excluded. In case PCOS is diagnosed, data will be used in the PCOS arm.

Inclusion criteria for participation are healthy women with a diagnosis of PCOS, healthy women without another diagnosis concerning the menstrual cycle, women aged between 18 and 40 years, and those with a stable heterosexual relationship for at least 6 months. Due to the nature of the erotic film material and the sexuality questionnaires, only heterosexual women are eligible.

Medical screening involves endocrine screening, assessment of BMI, a menstrual cycle history, a modified Ferriman-Gallwey score, and a transvaginal ultrasound in order to assess the ovarian morphology, antral follicle counts, and ovarian volumes of both ovaries. Total endometrial thickness will also be recorded as well as uterine sizes. The diagnosis of PCOS or of a normal menstrual cycle will be made after a standardized initial examination is performed between 07:45 and 10:00 am after an overnight fast in the early follicular phase of the menstrual cycle (third to fifth day of menses when no hormonal contraception is used) in case of a regular menstrual cycle. In case of a cycle irregularity, the examination will be performed on a random cycle day. In case of OCP use, screening will be on the eighth day of a new cycle after 7 days of not using OCPs. Endocrine features combined with medical screening will determine the diagnosis.

We will recruit women with PCOS and healthy women both either not using hormonal contraceptives (for at least 3 months prior to the start of their participation in this research project) or using OCPs for at least 3 months. We aim to include a similar number of women using OCPs as women not on OCPs in both the PCOS group and the control group.

Exclusion criteria for participation are psychiatric disorder, pregnancy or lactation, having undergone a hysterectomy (all indications), oophorectomy, or prolapse surgery, current or recent use of medications that are known to influence sexual response with the exception of OCPs, and (previous) medical disorders (other than PCOS) that are known to influence sexual response.

#### Sample Size Calculation

Considering the 5 primary outcome variables, we will apply Bonferroni correction, a 2-sided alpha of 0.01, and a power of 0.80.

ter Kuile et al reported differences between women with (N = 234) and without sexual problems (N = 108) on the FSFI and FSDS with effect sizes of, respectively, d = 1.9 and 2.3.^119^ For an effect size of d = 1.9, eight cases in each group are needed, and for an effect size of d = 2.3, 6 cases are required.

No data on the SDI are available for differences in women with PCOS. Strizzi et al^121^ reported an SDI of 38.1 (±16.56) in a sample of 29 women with a traumatic brain injury and a value of 49.59 (±21.09) in 29 normal controls, a difference of d = 0.60, suggesting that N = 67 cases for each group is required.

No data on VPA response in women with PCOS are available. However, there are VPA data available related to differences in premenopausal (N = 56) and postmenopausal (N = 32) women as reported by Both et al.^122^ In this study the difference in VPA response between premenopausal and postmenopausal women was reported as an F-value of 9.09 (degrees of freedom 1;84) corresponding to an effect size of n^2^ = 0.10 of d = 0.66. For such an effect 53 cases are needed in each group.

The largest sample needed is for the SDI; hence, we need 134 participants. Anticipating a small dropout of 10% we need 150 participants totally.

### Materials and Measures

#### Blood Samples and Endocrine Screening

Peripheral venous blood samples to assess androgen levels will be collected in all participants: total testosterone, SHBG, and calculated free androgen index: testosterone × 100/SHBG. Additionally, all of the following will be assessed in all participants: estradiol, progesterone, luteinizing hormone, follicle stimulating hormone, androstedione, dehydroepiandrosterone, dehydroepiandrosterone sulfate, thyroid stimulating hormone, prolactin, lipids, and fasting glucose and insulin. Plasma will be stored at −80°C. Samples will be analyzed using liquid chromatography mass spectrometry.

We will determine the number of CAG repeats in 4 different ways: the number of CAG repeats in the allele containing the fewest CAG repeats (short allele), the number of CAG repeats in the allele containing the most CAG repeats (long allele), the mean number of CAG repeats (biallelic mean), and the number of CAG repeats in the active X chromosome based on X-inactivation analysis (X-weighted biallelic mean). The CAG repeat region in the first exon of the AR gene on the X chromosome will be amplified from genomic DNA with a polymerase chain reaction-based assay. Another possibility is to define single nucleotide polymorphisms that will detect the different variable number tandem repeat alleles and perform a less cumbersome Global Screening Array. We probably will take the latter approach to determine AR sensitivity.

The medical and endocrine screening as well as the DNA analysis for all participants will be done at 1 laboratory at Erasmus University Medical Center, to ensure compatibility of data.

#### Questionnaires

We will measure the following variables as primary outcomes (Table 1): sexual function with the FSFI total score (19 items, cutoff <26.55 indicative of sexual dysfunction)^101^; sexual distress with the FSDS-R total score (13 items, score >15 indicative of distress)^101^; and solitary and dyadic sexual desire with the SDI (14 items, 2 scales [solitary desire scale and dyadic desire scale] and total scale; a higher score reflects higher levels of sexual desire)^102^ as an additional measure of sexual desire due to limitations in the desire scale of the FSFI.^103^

**Table 1:** Overview of primary and secondary outcome measures and independent variables

Outcome measures
Primary
Female Sexual Function Index
Female Sexual Distress Scale-Revised
Sexual Desire Inventory
VPA: genital sexual responsiveness
VPA: subjective sexual responsiveness
Secondary (mediators)
Sexual Esteem Scale
Rosenberg Self-esteem Scale
Body Image Self-consciousness Scale
The Multidimensional Body-Self-Relations Questionnaire-Appearance Scales
Hospital Anxiety and Depression Scale
Secondary (moderators)
Sexual and Physical Abuse Questionnaire
Sexual Excitation/Sexual Inhibition Inventory for Women
Revised Dyadic Adjustment Scale
Independent variables
PCOS status
OCP use

OCP = oral contraceptive pill; PCOS = polycystic ovary syndrome; VPA = vaginal pulse amplitude.

Other variables will be used as mediators (Table 1): sexual self-esteem measured by Sexual Esteem Scale (10 items, a higher total score indicates more sexual self-esteem)^49^^,^^104^; general self-esteem with the Rosenberg Self-Esteem Scale total score (10 items, a higher total score indicates more positive self-esteem)^105^, ^106^, ^107^; body self-consciousness with the Body Image Self-Consciousness Scale total score (15 items, a higher total score indicates greater body self-consciousness)^49^^,^^108^; body image with the Multidimensional Body-Self-Relations Questionnaire-Appearance Scales subscale (34 items, a higher total score indicates a more positive body image)^109^; and mental health with the Hospital Anxiety and Depression Scale total score (14 items, a higher total score indicates mental complaints).^110^^,^^111^

Some variables will be used as moderators (Table 1) as they are not caused by the independent variable (PCOS status): a history of sexual and physical abuse measured with Sexual and Physical Abuse Questionnaire (7 items, yes/no answers)^112^; sexual excitation and sexual inhibition propensity by Sexual Excitation/Sexual Inhibition Inventory for Women (36 items, 2 subscales, low Sexual Excitation Scale indicates low excitation, high Sexual Inhibition Scale indicates high inhibition)^113^; relationship satisfaction by Revised Dyadic Adjustment Scale total score (14 items, cutoff >48 for no distress).^114^^,^^115^

All questionnaires have been validated and will be available in a secure online environment.

#### Sexual Response Assessment

Sexual response will be assessed in 4 erotic stimulus conditions: 3-minute self-induced erotic fantasy, 2-minute vibrotactile glans clitoris stimulation, 5-minute erotic film, and 5-minute vibrotactile glans clitoris stimulation combined with an erotic film.^116^^,^^117^ Both erotic films depict heterosexual foreplay and intercourse. This film material has been previously used in studies from the same research group.^116^^,^^118^, ^119^, ^120^, ^121^ The short clips are taken from women friendly erotic films which are known to be effective in inducing a genital response. During the erotic fantasy condition, participants will be asked to fantasize about a pleasant sexual situation. The vibrotactile glans clitoris stimulation is provided by a small hands-off vibrator (2-cm diameter), attached to lycra panties. Vibrotactile glans clitoris stimulation has been used previously in studies on sexual response in women, and is known to elicit genital and subjective sexual arousal.^121^ The intensity of the vibration can be varied from very low^1^ to high.^10^ In the present study, high-intensity vibration will be provided. Each stimulus period is preceded by a 5-minute neutral film period, and followed by a 10-minute return-to-baseline interval during which participants will work on a non-erotic concentration task and watch a neutral film. A computer program coordinates the administration of the films and the vibrotactile glans clitoris stimulation.

All participants will go through the different stimulus conditions in the same order. Because sexual responses to erotic fantasy and vibrotactile glans clitoris stimulation are expected to differ according to androgen status, these 2 stimulus conditions are presented first, to avoid carry over from the visual stimuli that are known to be androgen independent and to elicit higher genital responses than fantasy and vibrotactile glans clitoris stimulation.^61^^,^^99^^,^^100^

Genital response will be measured using a vaginal photoplethysmograph assessing VPA.^122^, ^123^, ^124^ VPA is a sensitive, specific, and reliable measure of changes in vaginal vasocongestion in response to sexual stimulation^125^ and has been used in earlier studies in women with gynecological or neurological diseases.^120^^,^^126^ The vaginal device is sized and shaped as a vaginal tampon and can easily be inserted by the participant herself. It contains a light source and an optical sensor. The light source illuminates the capillary bed of the vaginal wall and the sensor responds to the light backscattered by the vaginal wall and the blood circulating within it. When the signal is connected to an alternating current amplifier, VPA is measured, which reflects the phasic changes in vaginal engorgement accompanying each heartbeat, with larger amplitudes reflecting higher levels of vaginal vasocongestion. Genital responses will be recorded continuously during the experiment. VPA will be averaged for the baseline condition and each erotic stimulus, and for each stimulus condition a change from the baseline score will be calculated. Ratings of self-reported sexual arousal and affect will be collected after each erotic stimulus. Participants will be asked to assess on a 7-point Likert scale their strongest feeling of sexual arousal.^127^^,^^128^

#### Procedure

**Figure 1:**
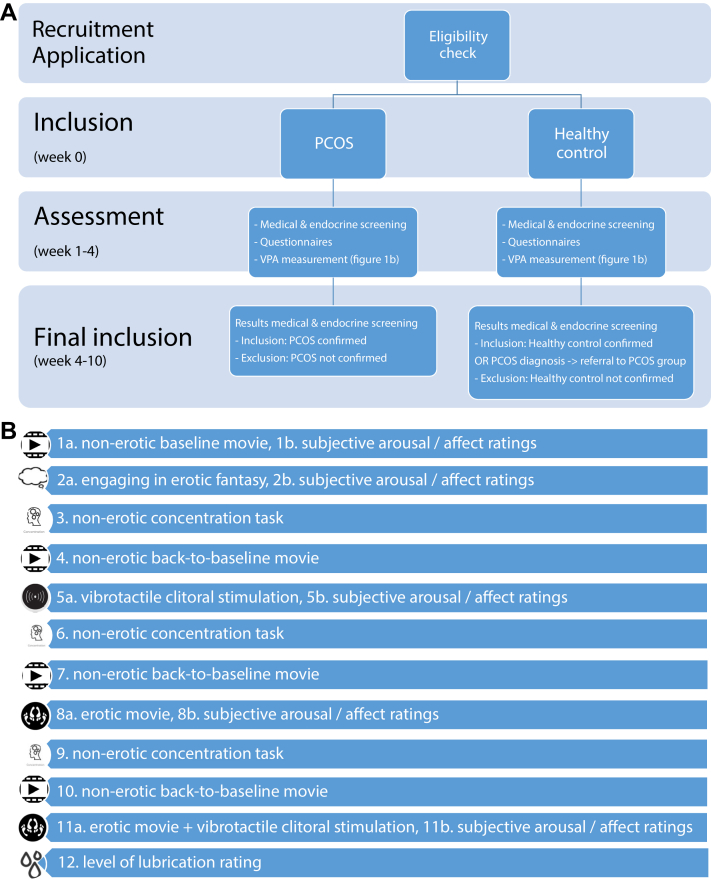
(A) Flowchart design protocol assessments. (B) Order of tasks and stimuli during VPA measurement. PCOS = polycystic ovary syndrome; VPA = vaginal pulse amplitude.

Women with PCOS and healthy control women will be asked to participate in the study and will be given an informational brochure. A week later a coordinator will contact each woman by telephone to give further information about the study, to answer questions, to check eligibility, and to determine participation. If a woman wants to participate, written informed consent will have to be provided. Only then peripheral venous blood samples will be collected (if not already recently done). After this the participant will receive an e-mail with a link to the web-based questionnaires. The questionnaires will also be available on paper if necessary. Within 2 weeks an appointment will be made to conduct the sexual response assessment (VPA) (Figure 1).

The sexual response assessment will be performed at the psychophysiological laboratory of LUMC or AMC. All women will be tested individually by a trained female experimenter. The experimenter will explain the details of the experimental procedure and the purpose of the experiment. The experimenter will then leave the room to allow the participant to apply the vibrator and insert the vaginal probe privately. The participant is allowed to put on her clothes again after applying the vibrator and inserting the vaginal probe. During the experimental procedure the experimenter will be in an adjacent room; contact will be possible by using an intercom. All further instructions, the stimuli, and the questionnaire about self-reported sexual arousal and affect will be presented on a monitor in the participant's room. At the end of the experiment, an exit interview questionnaire will be administered. Participants will be asked about their reactions to the experimental procedure and the use of the genital device (Figure 1).

### Statistical Analysis

Normality of the outcome data will be determined with Shapiro-Wilk tests. In case of non-normality, an appropriate transformation will be performed. The difference between women with PCOS and controls will be analyzed with separate multilevel regression analyses for each of the 5 outcomes. Independent variables will be PCOS (vs controls), fantasy (vs film), and vibration and interactions of PCOS with fantasy and vibration. *P* values of .01 will be considered significant.

For the differences between women with PCOS and controls, and women using OCPs and women not using OCPs, OCP use and interactions with PCOS, fantasy, and vibration will be added to the multilevel analyses.

Mediation regression analyses, as described by Mathieu and Tailor,^129^ will be extended to multilevel mediation regression analyses to estimate the mediating effects of steroid hormone levels, and sexual and psychosocial parameters in the relation of PCOS/OCP use on the 5 sexual outcome measures.

We will apply regression curve estimation with sexual function as the dependent variable and CAG repeat length and several functions of the CAG repeat length as independent variables to analyze a possible non-linear relation.

## Discussion

An important strength of this research project is the assessment of biological (eg, VPA, BMI, CAG repeats, extensive endocrine measurement) as well as psychosocial (eg, Hospital Anxiety and Depression Scale, Revised Dyadic Adjustment Scale) and sexual variables (eg, FSFI, FSDS-R) in women with and without PCOS. In so doing, we expect to be able to test a number of specific hypotheses with respect to sexual function in women with PCOS as well as to exploratively investigate associations between biopsychosocial variables and sexual function that may generate specific hypotheses for future studies.

Another strength of this study is the large number of participants including an age-matched control group. This large number of participants makes it possible to stratify the study according to PCOS phenotype or OCP use. This may help to further elucidate the role of OCP use and endocrine factors in sexual function of women without PCOS.

We encountered problems while including women with PCOS. We expected to include participants mainly through the Erasmus University Medical Center since the hospital has a PCOS expertise center and can therefore access a large population of women with PCOS. As it turned out, only few women recruited through this center were interested in or eligible to participate in this study. Recruitment through patient support groups worked very well and is ongoing. In hindsight, the difficulties in recruiting participants at the PCOS expertise center may be related to a large proportion of that patient population seeking medical treatment in order to become pregnant.

Also, the measurement of genital and subjective sexual response during exposure to sexual stimuli may have been a reason for non-participation. However, this did not complicate recruitment of control women and of women with PCOS recruited through patient support groups.

All participants were carefully recruited and assessed according to the study design.

Trial registration was retrospective but was done during the recruitment phase. Since this is not a clinical trial, registration was not required. We nevertheless consider trial registration and protocol publication of a cross sectional study such as the present one to be important, so as to enhance transparency about the design and hypotheses.

## Statement of authorship

Hester Pastoor: Design, Writing Protocol, Writing - Review & Editing; Stephanie Both: Design, Writing Protocol, Writing - Review & Editing; Reinier Timman: Methods And Statistical Section, Writing Protocol, Writing - Review & Editing; Ellen T.M. Laan: Design, Writing Protocol, Writing - Review & Editing; Joop S.E. Laven: Design, Writing Protocol, Writing - Review & Editing.
